# The parent and family impact of CLN3 disease: an observational survey-based study

**DOI:** 10.1186/s13023-024-03119-8

**Published:** 2024-03-18

**Authors:** Angela Schulz, Nita Patel, Jon J. Brudvig, Frank Stehr, Jill M. Weimer, Erika F. Augustine

**Affiliations:** 1 Department of Pediatrics, University Medical Center Hamburg-Eppendorf, Hamburg, Germany; 2 Amicus Therapeutics, Princeton, NJ USA; 3 Pediatrics & Rare Diseases Group, Sanford Research, Sioux Falls, SD USA; 4 NCL-Stiftung, Hamburg, Germany; 5 Kennedy Krieger Institute, Baltimore, MD USA

**Keywords:** Neuronal ceroid lipofuscinosis, Batten disease, Rare disease, Lysosomal storage disease, Juvenile NCL, Dementia, Quality of life

## Abstract

**Background:**

CLN3 disease (also known as CLN3 Batten disease or Juvenile Neuronal Ceroid Lipofuscinosis) is a rare pediatric neurodegenerative disorder caused by biallelic mutations in *CLN3*. While extensive efforts have been undertaken to understand CLN3 disease etiology, pathology, and clinical progression, little is known about the impact of CLN3 disease on parents and caregivers. Here, we investigated CLN3 disease progression, clinical care, and family experiences using semi-structured interviews with 39 parents of individuals with CLN3 disease. Analysis included response categorization by independent observers and quantitative methods.

**Results:**

Parents reported patterns of disease progression that aligned with previous reports. Insomnia and thought- and mood-related concerns were reported frequently. “Decline in visual acuity” was the first sign/symptom noticed by *n* = 28 parents (70%). A minority of parents reported “behavioral issues” (*n* = 5, 12.5%), “communication issues” (*n* = 3, 7.5%), “cognitive decline” (*n* = 1, 2.5%), or “seizures” (*n* = 1, 2.5%) as the first sign/symptom. The mean time from the first signs or symptoms to a diagnosis of CLN3 disease was 2.8 years (SD = 4.1). Misdiagnosis was common, being reported by *n* = 24 participants (55.8%). Diagnostic tests and treatments were closely aligned with observed symptoms. Desires for improved or stabilized vision (top therapeutic treatment concern for *n* = 14, 32.6%), cognition (*n* = 8, 18.6%), and mobility (*n* = 3, 7%) dominated parental concerns and wishes for therapeutic correction. Family impacts were common, with *n* = 34 (81%) of respondents reporting a financial impact on the family and *n* = 20 (46.5%) reporting marital strain related to the disease.

**Conclusions:**

Collectively, responses demonstrated clear patterns of disease progression, a strong desire for therapies to treat symptoms related to vision and cognition, and a powerful family impact driven by the unrelenting nature of disease progression.

## Background

CLN3 disease is a rare pediatric neurodegenerative disorder caused by biallelic pathogenic variants in the *CLN3* gene (OMIM 204,200). While disease onset and progression can vary depending on the causative variants, affected individuals typically present in early childhood with progressive vision loss followed in subsequent years by a range of neurological problems and deficits including seizures, motor dysfunction, dementia, and premature death. There are no approved treatments that cure the disease or slow disease progression, leaving affected individuals and their families with severe unmet need.

CLN3 disease is thought to be the most common form of Batten disease (also known as neuronal ceroid lipofuscinosis), a family of rare lysosomal disorders with characteristic accumulation of autofluoresent ceroid lipofuscin in lysosomes and predominant central nervous system presentation. Variants in at least 13 different genes lead to various forms of Batten disease with different patterns of onset and progression. Due to the rarity of each genetic form of Batten disease and the lack of diagnostic screening data, it is difficult to estimate precise incidence and prevalence. Collectively, Batten disease (as a group of diseases) has an incidence of approximately two to four individuals per 100,000 live births and represents the most prevalent pediatric neurodegenerative disorder [1].

The natural history of CLN3 disease progression has been well documented [2–11]. Age of onset ranges from four to seven years, and clinical presentation is typified by rapidly progressing vision loss [2]. Cognitive decline and behavioral problems begin shortly thereafter from seven to ten years of age, followed by seizures at ten to twelve years of age. Motor decline and parkinsonism progress over the second decade of life, leading to a loss of mobility. Throughout this progression, these symptoms require a complex blend of home care and hospital- or clinic-centered care, and even then serious unmet needs remain. Most patients succumb to the disease in the third or fourth decade of life. While this pattern of progression is followed by the vast majority of individuals with CLN3 disease, there is also a predominantly ophthalmic phenotype characterized by late-onset retinopathy alone, and there have been several reports of individuals with protracted disease course [12–16].

Extensive efforts have been undertaken to understand CLN3 disease etiology, pathology, and clinical progression; however, little is known about the impact of CLN3 disease on parents and caregivers. One study found evidence of an increased frequency of marital distress among 25 parents of individuals with CLN3 disease, with Dyadic Adjustment Scale scores indicative of marital distress in 28% of respondents, compared to an expected 16–20% in the general population [17]. Another recent study interviewed parents of five children with CLN3 disease, finding evidence of significant burdens on the family system related to feelings of loss, relational difficulties, and a lack of parental and caregiver support from the healthcare system [18]. A recent interview-based study examined parental support and problems in families of children with CLN3 disease, highlighting similar themes of care-related stress, strains on relationships, and healthcare-related challenges [19]. A similar study explored the challenges faced by individuals and families living with CLN2 disease, demonstrating significant reductions in health-related quality of life, increases in stress and sleep disruption, and substantial financial burden [20].

While the family impact of CLN3 and other forms of Batten disease is immense, many aspects of the parental and family experience remain elusive. For example, what experiences are typical of the diagnostic journey? How does CLN3 disease influence the affected individual’s life experience? For which symptoms do parents most desire treatment? How has CLN3 disease impacted family decisions related to work, marriage, and finances? We investigated these questions using semi-structured interviews with parents of individuals with CLN3 disease along with response categorization by independent observers and quantitative analysis.

## Methods

The study was approved by the Western Institutional Review Board (IRB Tracking Number 20210943). The objective of the study was to increase the understanding of the burden of disease in affected individuals diagnosed with CLN3 Batten disease and in their families. Study design and questions were developed with input from patient advocacy organizations related to Batten disease including the Batten Disease Support and Research Association (BDSRA) Foundation, as well as caregivers and healthcare providers.

### Consent and recruitment

Participants were recruited from countries where English or German are commonly spoken, including Australia, Canada, Denmark, Germany, Norway, Sweden, the United Kingdom, and the United States. English or German language recruitment materials were distributed by patient advocacy organizations BDSRA Foundation, Beyond Batten Disease Foundation, Batten Disease Family Association, NCL Sweden, NCL Denmark, NCL-Gruppe Deutschland, and other Batten disease-related organizations, as well as Engage Health’s EnCompass® database (an opt-in database of rare disease patients).

Inclusion criteria were being a parent or legal guardian of a person with CLN3 disease; diagnosis confirmed with proof of CLN3 disease diagnosis; parent/legal guardian age ≥ 18 years at the time of consent; ability to read, write, and communicate in English or German; ability to grant informed consent; willingness to complete a survey; willingness to participate in a 50-minute telephone interview; and ability to view or receive a document from the interviewer before or during the interview.

All respondents voluntarily agreed to participate and provided written informed consent along with proof of CLN3 disease diagnosis (e.g., genetic testing results) and demographic information. Aside from proof of disease diagnosis, no medical records were reviewed as part of this study.

### Interviews

Consented participants each engaged in a 50-minute telephone interview conducted by a trained interviewer. Prior to study initiation, interviewers were trained in qualitative research methods, interviewing skills, interpersonal skills, and study procedures. An interview guide was developed and validated prior to the study. This guide was utilized by interviewers during the study/interviews to ensure consistent and productive conversations. Interviews were conducted by Engage Health (Eagan, MN). Interviewers asked open-ended questions about the signs and symptoms of CLN3 disease, the path to diagnosis and disease progression, healthcare use, and the impact of the disease on the affected individual and their family. During the call, summarized responses were entered in real time into a database by the interviewer and verbally verified with the participant to confirm accuracy. Participants were free to skip any question as desired or to terminate the interview at any time. Interviews were recorded if participants agreed to recording. Accuracy of transcribed and codified responses was confirmed with scheduled and random audits by a senior qualitative researcher that compared voice recordings with database entries.

### Data analysis

The open-ended questions were coded by two independent coders to generate quantitative data on frequency of various response types. Coders were trained in qualitative research methods and coding experience was developed and validated with parallel coding alongside an experienced coder prior to engaging in independent coding. If there was disagreement between the two coders for any data point, they met to review the participant comments together in an attempt to reach agreement. If agreement was still not reached, an experienced third party was consulted. For analysis of medications prescribed, brand and drug names were combined as appropriate. Participants were asked to report any medications related to CLN3 disease, used in the past or present. Where frequencies are reported, they reflect percentage based on the number of respondents to each specific item. Where ages are reported for diagnosis or symptom onset, if a range of ages was given, the midpoint was used. For ambiguous responses (e.g., “almost eight”), the nearest whole number age was chosen. Where participants were asked to report answers on a scale of one to ten, a simple numeric rating scale was used, with no definitions other than ten being highest. Descriptive statistics on demographic data, temporal data, and frequency of responses were analyzed by Metrics for Learning (Maricopa, AZ; USA) with Microsoft Excel and Statistics Package for the Social Sciences (SPSS).

## Results

### Demographics

To learn more about the parent and family impact of CLN3 disease, we developed and utilized an 85- question, semi-structured survey focusing on the observed signs and symptoms of the disease, the path to diagnosis, disease progression, healthcare experiences, and the impact of the disease on affected individuals and their families. Target participants were individuals living with CLN3 disease and parents/caregivers of individuals with CLN3 disease. During the telephone-conducted surveys, trained interviewers asked participants closed and open-ended questions and recorded summarized answers in a database. Some responses were later coded by independent observers to categorize various types of responses for quantitative analysis.

Thirty-nine individuals agreed to participate, all of whom were parents (27 mothers, 7 fathers, 5 with unreported parental gender identity) of individuals with a genetic diagnosis of CLN3 disease (Table 1). Thirty-five parents had one child with CLN3 disease, while four parents had two children with CLN3 disease. Affected individuals resided in North America (*n* = 16), Europe (*n* = 26), and Australia (*n* = 1). At the time of the survey, 38 of the affected individuals were alive, while five had succumbed to their disease. Twenty-four of the affected individuals were male, while nineteen were female.

**Table 1:** Participant demographics

**Survey participants, ** *n*	39							
**Relationship to affected individual(s), ***n*** (%)**	**Mother** 27			**Father** 7		**No Response** 5		
**Number of affected children in family**	**One** 35 (89.7%)			**Two** 4 (10.3%)				
**Number of unaffected children in family**	**Zero** 9 (23.1%)	**One** 14 (35.9%)	**Two** 9 (23.1%)		**Three** 5 (12.8%)		**Four** 1 (2.6%)	**Five** 1 (2.6%)
**Continent of residence, n (%)**	**North America** 16			**Europe** 26		**Australia** 1		
**Affected individuals, ** *n*	43							
**Present age (if surviving, ***n*** = 38), mean years (SD, range)**	12.4 (5.5, 2.4–26.1)							
**Age at death (if deceased, ***n*** = 5), mean years (SD, range)**	23.1 (6.9, 15.0-33.2)							
**Sex, ***n*** (%)**	**Male** 24 (55.8%)			**Female** 19 (44.2%)				

### Diagnosis and symptomatic progression

Reported diagnosis timing and symptomatic progression closely followed patterns reported in the literature (Table 2) [2]. Forty (93.0%) individuals were symptomatic at diagnosis, presenting with vision symptoms, while the remainder were diagnosed following genetic testing in the absence of symptoms (i.e., following the diagnosis of a relative). The mean age at diagnosis was 8.2 years. “Decline in visual acuity” was the first sign/symptom noticed by 28 parents (70% of the parents who responded to the question), with a minority of parents reporting “behavioral issues” (*n* = 5, 12.5%), “communication issues” (*n* = 3, 7.5%), “cognitive decline” (*n* = 1, 2.5%), or “seizures” (*n* = 1, 2.5%) as the first sign/symptom. Similarly, “decline in visual acuity” was the second sign/symptom noticed by six parents (*n* = 6, 46.2%), with less frequent reports of “behavioral issues” (*n* = 3, 23.1%), “communication issues” (*n* = 2, 15.4%), or “cognitive decline” (*n* = 2, 15.4%). The third sign/symptom noticed by parents was “decline in visual acuity” (*n* = 3, 60.0%), followed by “cognitive decline” (*n* = 1, 20%), and “gross motor delay” (*n* = 1, 20%). “Seizures” were noted as the fourth presenting sign/symptom by two parents.

**Table 2:** Diagnosis and progression of symptoms

**Age at diagnosis (** *n* ** = 43), mean years (SD)**	8.2 (4.0)			
**Symptomatic at diagnosis? ** *n* ** (%)**	**Yes** 40 (93.0%)	**No** 3 (7.0%)		
**Initial signs/symptoms noticed by caregiver**	**First** **(** *n* **= 40)**	**Second** **(** *n* **= 13)**	**Third** **(** *n* **= 5)**	**Fourth** **(** *n* **= 2)**
	Decline in visual acuity 28 (70.0%)	Decline in visual acuity 6 (46.2%)	Decline in visual acuity 3 (60.0%)	Seizures 2 (100.0%)
	Behavioral issues 5 (12.5%)	Behavioral issues 3 (23.1%)	Cognitive decline 1 (20.0%)	
	Communication issues 3 (7.5%)	Communication issues 2 (15.4%)	Gross motor delay 1 (20.0%)	
	Cognitive decline 1 (2.5%)	Cognitive decline 2 (15.4%)		
	Seizures 1 (2.5%)			
**Symptom prevalence and onset (years)**	**Symptom present?**	**Mean age of onset, years (SD)**		
**Decline in visual acuity**	41 (95.3%)	5.7 (1.2)		
**Insomnia**	37 (86.0%)	7.4 (3.6)		
**Learning difficulties**	33 (76.7%)	8.3 (4.1)		
**Behavioral problems**	32 (74.4%)	6.3 (4.1)		
**Memory loss**	32 (74.4%)	9.7 (4.0)		
**Anxiety**	30 (69.8%)	9.7 (5.9)		
**Seizures**	28 (65.1%)	10.5 (3.5)		
**OCD**	28 (65.1%)	8.1 (3.9)		
**Ataxia**	24 (55.8%)	12.4 (3.6)		
**Depression**	17 (39.5%)	11.7 (5.7)		
**Missed milestones at checkups**	13 (30.2%)	3.3 (2.3)		

**Fig. 1:**
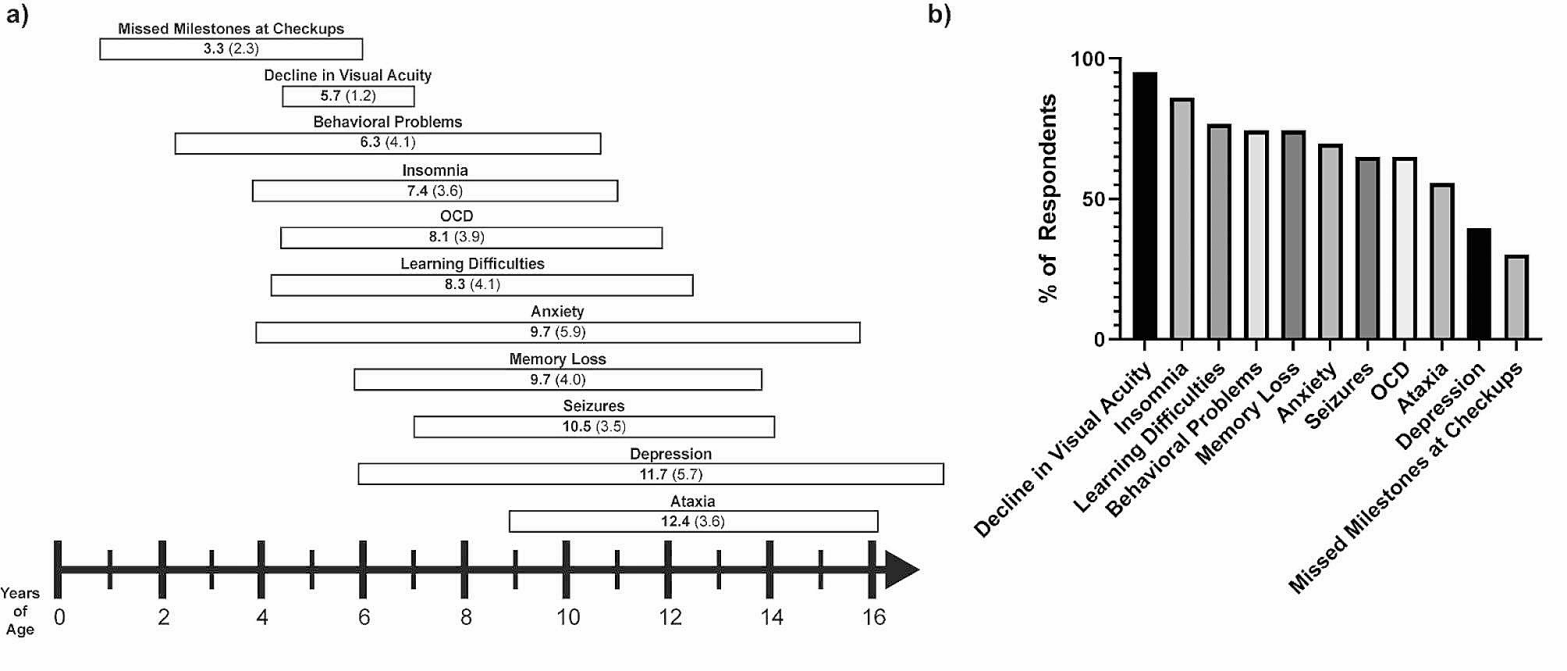
Symptom onset and prevalence. **(a)** Timeline showing caregiver-reporter timing of symptom onset for individuals with CLN3 disease, sorted from earliest to latest onset. Mean onset is in bold, with standard deviation in parentheses. **(b)** Percent of respondents indicating that each sign or symptom was ever present, sorted from most to least frequent

Parents were also asked about the approximate age of onset for any presenting symptoms, and mean age of onset and symptom penetrance (%) was calculated based on these data (Fig. 1). Some parents (30.2%) noted “missed milestones at checkups” occurring early in life, with a mean age of onset of 3.3 years. “Decline in visual acuity” had the highest penetrance at 95.3%, with a mean age of onset of 5.7 years. Behavioral problems began around the same time or shortly thereafter (74.4%), with a mean age of onset of 6.3 years. “Insomnia” was noted with very high frequency (86%) with a mean age of onset of 7.4 years. Several other symptoms were noted with high or moderate frequency including “learning difficulties” (76.7%, mean age of onset 8.3 years), “memory loss” (74.4%, mean age of onset 9.7 years), “anxiety” (69.8%, mean age of onset 9.7 years), “obsessive-compulsive disorder (OCD)” (65.1%, mean age of onset 8.1 years), “ataxia” (55.8%, mean age of onset 12.4 years), and “depression” (39.5%, mean age of onset 11.7 years).

### Clinical care

Participants were also asked about the affected individual’s experiences with diagnosis, clinical tests, and medications (Table 3). The mean time from the first signs or symptoms to a diagnosis of CLN3 disease was 2.8 years. Misdiagnoses were frequent, being reported by 24 participants (55.8%). The most common misdiagnoses were retinitis pigmentosa (also known as retinopathia pigmentosa) (*n* = 7, 16.3%) and rod/cone dystrophy (*n* = 7, 16.3%), followed by Stargardt disease (*n* = 5, 11.6%), autism (*n* = 4, 9.3%), schizophrenia (*n* = 2, 4.7%), and a range of other misdiagnoses (all *n* = 1, 2.3% each). Following accurate diagnosis, most individuals had eye exams (*n* = 23, 53.5%), electroencephalograms (EEGs, *n* = 23, 53.5%), and/or magnetic resonance imaging (MRI, *n* = 23, 53.5%). A minority of participants also reported bloodwork (*n* = 9, 20.9%), computed tomography scans (CT scans, *n* = 7, 16.3%), and/or genetic tests (*n* = 5, 11.6%). Affected individuals were prescribed an average of 7.6 medications related to the disease over time (SD = 12.9) encompassing a wide range of indications and drug classes. The most commonly prescribed medications were melatonin (*n* = 17, 39.5%), levetiracetam (*n* = 11, 25.6%), valproic acid (*n* = 11, 25.6%), lamotrigine (*n* = 9, 21.0%), clobazam (*n* = 8, 18.6%), clonazepam (*n* = 9, 16.3%), and midazolam (*n* = 7, 16.3%).

**Table 3:** Clinical care

**Time from first signs/symptoms to CLN3 disease diagnosis, mean years (SD)**	2.8 (4.1)		
**Was a misdiagnosis ever given?**	**Yes** 24 (55.8%)	**No** 19 (44.2%)	
**Most frequent misdiagnoses**	Retinitis pigmentosa		7 (16.3%)
	Rod/cone dystrophy		7 (16.3%)
	Stargardt disease		5 (11.6%)
	Autism		4 (9.3%)
	Schizophrenia		2 (4.7%)
	Other (Meningitis, optic atrophy, sensory processing disorder, oppositional defiant disorder, epilepsy, mitochondrial disease, anxiety, Bardet Biedl syndrome)		1 each (2.3%)
**Clinical tests performed after diagnosis**	Eye exam		23 (53.5%)
	EEG		23 (53.5%)
	MRI		23 (53.5%)
	Bloodwork		9 (20.9%)
	CT scan		7 (16.3%)
	Genetic test		5 (11.6%)
**Medications (related to CLN3 disease) used by ≥ 2 affected individuals**	melatonin		17 (39.5%)
	levetiracetam		11 (25.6%)
	valproic acid		11 (25.6%)
	lamotrigine		9 (21.0%)
	clobazam		8 (18.6%)
	clonazepam, midazolam		7 (16.3%)
	lorazepam		6 (14.0%)
	quetiapine, sertraline, polyethylene glycol 3350 laxative		5 (11.6%)
	diazepam, trazodone, zonisamide		4 (9.3%)
	aripiprazole, lorazepam, baclofen, clonidine, divalproex sodium, ibuprofen, midazolam, fluoxetine, sertraline, vitamin D		3 (7.0%)
	albuterol, cyproheptadine, domperidone, fluticasone, gabapentin, Gaviscon®, hydroxyzine, pregabalin, methadone, morphine, multivitamin, omeprazole, paracetamol, phenobarbital, risperidone, topiramate, trazadone, ondansetron		2 (4.7%)

### Impact on life activities

Parents were asked questions about the impact of the disease on their diagnosed children (Table 4). First, parents were asked, “What is the most important thing you wish your child could do but can’t because of CLN3 disease?” The most common category of responses related to “decline in visual acuity” (*n* = 16, 37.2%); followed by general wishes related to living a “normal life” (*n* = 5, 11.6%); or a “normal life” as it relates to “sports and leisure” (*n* = 5, 11.6%), “cognition” (*n* = 4, 9.3%), “socialization” (*n* = 2, 4.7%), “socialization/travel” (*n* = 1, 2.3%), “lifespan” (*n* = 1, 2.3%), “reading” (*n* = 1, 2.3%), or “lifespan/mobility” (*n* = 1, 2.3%). Two participants (4.7% each) expressed wishes related to “expressive communication,” while one participant (2.3% each) expressed wishes for either “career aspirations,” “cognition,” “coping skills– mood,” being “treated normally,” or “play and leisure.” Parents’ wishes seemed to reflect the evolving impact of different symptoms as the disease progressed; parents of younger individuals (≤ 18 years of age, *n* = 33) were more likely to list “visual acuity” as their greatest wish (*n* = 15, 45.5%), while none of the parents of older (> 18 years of age, *n* = 5) or deceased individuals (*n* = 5) listed this wish, instead describing wishes relating to a “normal life” (*n* = 8, 80%), “career aspirations” (*n* = 1, 10%), or “communication” (*n* = 1, 10%).

**Table 4:** Life impact of symptoms

**What is the most important thing you wish your child could do, but can’t because of CLN3 disease?**	**First** **(** *n* **= 43)**		**Second** **(** *n* **= 41)**	
	Decline in visual acuity 16 (37.2%)		“Normal life” – sports and leisure 7 (17.1%)	
	“Normal life” 5 (11.6%)		Normal cognition for age 5 (12.2%)	
	“Normal life” – sports and leisure 5 (11.6%)		Expressive communication, independence, “normal life” – socialization 4 each (9.8%)	
	“Normal life” – cognition 4 (9.3%)		Mental health, mobility, “normal life” – interpersonal relationships, decline in visual acuity 2 each (4.9%)	
	Expressive communication, “normal life” – socialization 2 each, (4.7%)		Family support, life without seizures, longer lifespan, mood regulation, “normal life” – independence, “normal life”– physical decline, “normal life” – stamina 1 each (2.4%)	
	Career aspirations, cognition, coping skills – mood, “normal life” – socialization/travel, “normal life” – lifespan, “normal life” – reading, “normal life” – lifespan/mobility, treated normally, play and leisure 1 each (2.3%)			
**What is the most important issue that you would like to see treated by a therapy?**	**First** **(** *n* **= 43)**	**Second** **(** *n* **= 43)**		**Third** **(** *n* **= 40)**
	Maintain/improve vision 14 (32.6%)	Maintain/improve cognition 11 (25.6%)		Maintain/improve vision 9 (22.5%)
	Stop disease progression 9 (20.9%)	Maintain/improve vision 10 (23.3%)		Maintain/improve mobility 7 (17.5%)
	Maintain/improve cognition 8 (18.6%)	Maintain/improve mobility 6 (14.0%)		Maintain/improve cognition 6 (15.0%)
	Eliminate/reduce epilepsy/seizures, maintain/improve mobility 3 each (7.0%)	Eliminate/reduce epilepsy/seizures 5 (11.6%)		Maintain improve speech 5 (12.5%)
	Slow disease progression 2 (4.7%)	Maintain/improve speech 3 (7.0%)		Stop/reverse disease progression 4 (10%)
	Improve speech, improve state of mind – loneliness and frustration, increase lifespan, prevent/improve anxiety attacks 1 each (2.3%)	Increase lifespan, slow disease progression 2 each (4.7%)		Improve behavior, eliminate/reduce epilepsy/seizures 2 each (5%)
		Improve independence, provide hope, reduce/eliminate tremors 1 each (2.3%)		Provide hope, improve mood, improve sleep, prevent disease, provision of respite care 1 each (2.5%)

Parents were also asked, “What is the most important issue that you would like to see treated by a therapy?” The most common response categorization was “maintain/improve vision” (*n* = 14, 32.6%), followed by “stop disease progression” (*n* = 9, 20.9%), “maintain/improve cognition” (*n* = 8, 18.6%), “eliminate/reduce epilepsy/seizures” (*n* = 3, 7.0%), “maintain/improve mobility” (*n* = 3, 7.0%), “slow disease progression” (*n* = 2, 4.7%), “improve speech” (*n* = 1, 2.3%), “improve state of mind– loneliness and frustration” (*n* = 1, 2.3%), “increase lifespan” (*n* = 1, 2.3%), and “prevent/improve anxiety attacks” (*n* = 1, 2.3%). Parents were also asked about the second and third most important issues that they would like to see treated by a therapy. Here, too, the most common responses related to “maintain/improve vision” (second most important *n* = 10, 23.3%; third most important *n* = 9, 22.5%), or “maintain/improve cognition” (second most important *n* = 11, 25.6%; third most important *n* = 6, 15%). “Maintain/improve mobility” was also frequently mentioned as a second most important issue (*n* = 6, 14%) or third most important issue (*n* = 7, 17.5%).

### Family impact

Finally, parents were asked questions related to the impact of the disease on family finances and work decisions (Table 5). Thirty-four parents (81.0%) stated that CLN3 disease had a financial impact on their family and rated the degree of that impact at a mean of 5.6 on a scale of one to ten. Parents reported that a mean of 13.0% of their family income went to CLN3-related expenses. Parents also reported that a variety of life decisions had to be made based on their child’s CLN3 disease. These included working fewer hours (*n* = 24, 55.8%); not working, leaving work, losing jobs, or deciding not to work (*n* = 16, 37.2%); and influencing the type of career that they pursued (*n* = 9, 20.9%). Parents also reported marital strain related to CLN3 disease (*n* = 20, 46.5%), deciding not to have more children (*n* = 17, 39.5%), and CLN3 disease being a factor in a divorce or separation (*n* = 4, 9.3%). Despite these challenges, twelve parents (27.9%) also reported that their family had been brought closer because of CLN3 disease.

**Table 5:** Family impact

**Has CLN3 disease had a financial impact on your family (** *n* ** = 42)?**	Yes	34 (81.0%)
	No	8 (19.0%)
**On a scale of 1 to 10 (highest impact), how much impact has CLN3 disease had on your family’s financial situation? mean (SD)**	5.6 (3.0)	
**What percent of your family income would you estimate CLN3-related expenses represent? mean (SD)**	13.0 (17.5)	
**What life decisions have you (the caregiver) made based on your family member’s CLN3 disease (** *n* ** = 43)?**	**Work & Career**	
	Work fewer hours	24 (55.8%)
	Cannot work, left work, lost job, or decided not to work	16 (37.2%)
	Influenced type of career	9 (20.9%)
	**Marriage & Family**	
	Marital strain impacted related to CLN3 disease	20 (46.5%)
	Decided not to have more children	17 (39.5%)
	Family brought closer by CLN3 disease	12 (27.9%)
	CLN3 disease was a factor in divorce/separation	4 (9.3%)

## Discussion

Here, we present one of the largest CLN3 caregiver surveys to date, which includes responses from 39 parents of individuals with CLN3 disease. We analyzed responses on a broad range of topics including the observed signs and symptoms of the disease, the path to diagnosis, healthcare experiences, and the impact of the disease on affected individuals and their families. The responses reflect the persistent struggles faced by individuals with the disease and their families and shed new light on some of the most impactful aspects of the disease.

Despite some heterogeneity and phenotypic variability in the clinical manifestations of CLN3 disease reported in the literature (particularly for rare alleles) [12–15], the order of symptom onset follows a predictable course of progression. CLN3 disease is known to present with rapidly progressing vision loss, followed by broader neurological involvement including cognitive changes and seizures, eventually leading to loss of motor function and ambulation [2]. Indeed, a recently described clinical staging system for CLN3 disease relies entirely on the presence or absence of these three classes of symptoms [21]. In the present study, while parents’ descriptions of disease onset and progression largely recapitulate the patterns described in the literature, they also suggest other common, perhaps underreported, features of the disease.

As expected, decline in visual acuity was the symptom with the greatest penetrance, reported by 95.3% of respondents. Somewhat surprisingly, insomnia was the symptom with the next greatest penetrance, reported by 86.0% of respondents. Parents recounted various sleep issues related to circadian schedules, frequent waking, and night terrors. The high prevalence of sleep issues was also reflected in responses related to medication usage, where melatonin was reported with the highest frequency (39.5%) among all medications. While sleep issues are known sequela of both blindness and dementia and have been previously reported in CLN3 disease, the very high prevalence reported here suggests that it may be a characteristic aspect of the disease that potentially contributes to other cognitive and behavioral problems [22–24].

While a range of cognitive and behavioral issues have long been described in CLN3 disease, the results here provide the first parent-reported prevalence data for some of these challenging symptoms. Generalized “behavior problems” were described as occurring early in disease progression (mean onset 6.3 years of age) and with high prevalence (74.4%). More specifically, parents also noted a high prevalence of symptoms relating to “OCD” (65.1%), “anxiety” (69.8%), and “depression” (39.5%). While it is unclear how many of these responses reflect formal psychiatric diagnoses, their high prevalence is indicative of a common perception among parents that their CLN3-affected children exhibit thought- and mood-related issues. Parents emphasized recurring patterns of obsessive behaviors and thoughts, rigid adherence to routines, intense fear or anxiety particularly in new situations, and depressed mood (often relating to social isolation). These responses recalled those of prior studies that have described many affected individuals as “routine-bound, ritualistic, and engaged in repetitive questioning or discussions” [25]. Some parents temporally or causally linked these issues directly to other symptoms (e.g., anxiety as a consequence of blindness or dementia), as reported in prior studies [19]. Other parents believed them to be unique and distinctive aspects of the disease. Collectively, the symptomatic prevalence responses reported here reinforced the multifaceted nature of the physical, behavioral, and cognitive challenges facing families affected by the disease, while also highlighting the unique constellation of symptoms that distinguish CLN3 disease from other differential diagnoses.

Parents’ wishes for therapeutic correction closely mirrored the symptomatic prevalence responses. “Maintain/improve vision” was the top issue a plurality of parents desired to see improved by a therapy (32.6%) followed by general halting of “disease progression” (20.9%) and improvement of cognition (18.6%). Vision and cognition were also high in the list of second and third priorities, along with “maintain/improve mobility.” Fortunately, these groups of symptoms mirror those targeted by developers of therapeutics, which are testing gene therapies that target the brain and/or retina [26–28], ASOs (antisense oligonucleotide) broadly targeting the CNS [29], or small molecule therapies intended for systemic treatment [30, 31]. It is also likely that current and upcoming clinical trials will report on these types of outcomes, as the most well-validated clinical tools for CLN3 disease (e.g., the United Batten Disease Rating Scale (UBDRS); the Vineland Adaptive Behavior Scales) contain domains related specifically to vision, physical, and cognitive impairment [4, 5, 11]. Parents’ wishes for therapeutic correction also reinforce the ongoing need for effective palliative care strategies to assist with symptoms related to mobility, seizures, and emotional concerns.

Responses also highlighted the immense financial and family impact of CLN3 disease, another area with an ongoing need for support. Parents commonly made choices related to work hours, career type, or leaving the workforce because of the disease. Family strain related to the disease was also evident, with frequent reports of CLN3-related sibling impact, marital strain, and family planning decisions. These responses reiterate some of the difficulties highlighted in a prior survey-based study of parents of CLN3-affected individuals, which reported a high frequency of conflicts related to time commitments, stress on family systems, and strained romantic relationships [18]. On the other hand, both the present study and prior work have also found that the difficulties imposed by the disease frequently resulted in adaptations described in a positive light by parents [18]. Here, one frequently reported family impact was a sense of having been brought closer by the disease (27.9%).

This study had several limitations worth noting. Recruitment was primarily through patient advocacy foundations, which may bias the sample towards families that are well connected to such support. Several potentially helpful data points (e.g., specific milestones missed at checkups, impacts on unaffected siblings, etc.) were not collected in the surveys, which aimed to balance exhaustiveness with the potential for survey fatigue. Additionally, the limited participant numbers (reflective of the rareness of CLN3 disease), preclude the ability to conduct certain analyses, such as differences in responses stratified by family size or geography, with appropriate statistical power.

## Conclusions

This work adds new insights to the body of evidence documenting family and parental experiences with CLN3 Batten disease while also highlighting the vast unmet need that remains. In most cases, disease progression is rapid and unrelenting; just as families adapt to existing implications of the disease they are faced with new ones. As disability builds, so do the financial and emotional resources required for care. Healthcare providers assist where they can but have few tools at their disposal that can slow or stabilize disease progression. As the biological functions of the CLN3 protein are finally being revealed [32–34], new insights could inform new approaches for therapeutic development and provide hope for the affected families. Clinical trials are already underway, testing new therapies including a gene therapy aiming to restore CLN3 expression and function throughout the nervous system [35, 36] (clinicaltrials.gov identifiers NCT03770572 and NCT05174039). In the coming years, families will hopefully be offered effective treatments that address their core concerns.

## Data Availability

The full survey response datasets used in this study are not openly available in order to protect participant privacy, but deidentified datasets are available from the corresponding author upon reasonable request.
